# The incidence of preeclampsia in ICSI pregnancies 

**DOI:** 10.12669/pjms.301.3982

**Published:** 2014

**Authors:** BurcuArtunc Ulkumen, DilekBenk Silfeler, Kenan Sofuoglu, Ibrahim Silfeler, Vedat Dayicioglu

**Affiliations:** 1 Burcu Artunc Ulkumen, Department of Obstetrics and Gynecology, Celal Bayar University, Faculty of Medicine, Turkey.; 2 DilekBenk Silfeler, Department of Obstetrics and Gynecology, Mustafa Kemal University, Faculty of Medicine, Antakya/Hatay, Turkey.; 3 Kenan Sofuoglu, Department of Obstetrics and Gynecology, ZeynepKamil Education and Research Hospital, Turkey; 4 Ibrahim Silfeler, Department of Pediatrics, Mustafa Kemal University, Faculty of Medicine, Antakya/Hatay, Turkey.; 5 Vedat Dayicioglu, Department of Obstetrics and Gynecology, ZeynepKamil Education and Research Hospital, Turkey

**Keywords:** Preeclampsia, Etiology, Paternal factor, Infertility

## Abstract

***Objective: ***We aimed to evaluate the association between infertility etiology in ICSI pregnancies and preeclampsia; besides, we aimed to discuss the effect of the paternal factor in the pathogenesis of preeclampsia.

***Hypothesis:***We hypothesized that preeclampsia is more common in ICSI pregnancies with male factor. It is known that maternal exposure to paternal sperm cells over a time period has a protective effect against preeclampsia. Male partners with azospermia have no sperm cells in their seminal fluid, whose female partners will not be able to develop some protective immunity against preeclampsia. We hypothesized that the infertile couples with male factor (partner with azoospermia and also oligospermia) would be an ideal model to test the partner-specific protective immunity against preeclampsia, as the women had no chance to develop adequate protective immunity via the partner’s sperm exposure.

***Methods: ***This Single-center, retrospective study included 508 infertile couples admitted to our IVF center between January 2001 and March 2008. The data regarding the maternal age, etiology of the infertility, the pregnancy rates, abortus ratio and viable pregnancy rates was collected from the case files. Antenatal complications such as preeclampsia, placenta previa, abruptio placenta, premature rupture of membranes, premature labor, oligohydramnios, gestational diabetes, postmaturity, postpartum complications and neonatal outcomes were evaluated via the file records and phone interviewing. The study population was divided into two main groups according to the etiology of infertility. 301 of the study population (group 1) was infertile due to male factor and 207 of the study population (group 2) was female factor and unexplained infertility cases.Group 1 patients were divided further into two subgroups: group 1a included 56 cases in which TESE (testicular sperm extraction) was used to obtain the sperm cells as the male factor was severe and as there was no sperm cells in seminal fluid. Group 1 b consists of 245oligospermic cases who obtained sperm cells via conventional methods.

***Results: ***The mean ages of women in Group one and two were 30.22±5.06 and 31.58±4.36 years respectively (p=0.001). 129 cases (42,8%) from group one and 106 cases (51,2%) from Group two ended in first trimester and early second trimester (<24 gestational weeks) pregnancy loss. In group one, only 172 cases of 301 pregnancies passed over 24 weeks of gestational age, whereas in group two, 101 cases of 207 patients passed over 24 gestational weeks. There was no significant difference between two groups regarding chemical pregnancies and early pregnancy loss (p=0.314). There was no significant difference between the groups regarding placenta previa, gestational diabetes, oligo hydramnios and intrauterine growth retardation. One one pregnancy was 1.5 times more vulnerable for preeclampsia.

***Conclusion: ***Pregnancies with azoospermic and oligospermic partners had an increased risk for developing preeclampsia.

## INTRODUCTION

Hypertension in pregnancy is a disease unique for human beings. According to the selected population and diagnostic criteria, its incidence varies between 2% to 7%.^1^^,^^2^

Preeclampsia is still one of the major causes of maternal mortality in developed countries with an incidence of 15%-20%.^3^^,^^4^ Furthermore, it is also one of the main causes of neonatal mortality and morbidity, directly as it causes intrauterine growth retardation and indirectly as it is often seen together with placental abruption. Annually, 50000 women and 900000 newborn in the world die because of this disease.^5^

Regarding the etio-pathogenesis of preeclampsia, it was thought that only the maternal cardiovascular and endothelial systems which could not overcome normal pregnant physiology were responsible for the disease, as preeclampsia is the result of complex generalized endothelial dysfunction. Nevertheless, recent studies have pointed that not only maternal improper physiological response but also paternal effect had an important role in the pathogenesis.^2^

Epidemiological studies have showed that maternal exposure to paternal sperm cells had protective effect against preeclampsia. The association between maternal sperm exposure and preeclampsia became apparent with the higher incidence of preeclampsia in women with shorter duration of preconceptional sperm exposure.^8^Adolescent pregnancies with higher preeclampsia risk may also be the result of the short duration of the maternal sperm exposure. Furthermore, preeclampsia is higher among women using barrier contraceptive methods which prevent sperm exposure and alloimmunisation.^9^ Furthermore, maternal sperm exposure induces an inflammatory response via decidual secretion of T- helper 1, cytokines, proteolytic enzymes and free radicals.^6^^,^^7^ This inflammation is needed for the proper implantation of the embryo into the uterus. 

A previously healthy pregnancy is protective against preeclampsia, however this protective effect is lost with partner change.^9^^,^^10^ This negative effect of changing paternity promotes the possible immunologic contribution of the partner for the pathogenesis of preeclampsia. These results are compatible with hypothesis that sperm exposure has a protective role against preeclampsia.

From that point of view, we hypothesized that infertile couples with male factor would have increased risk for preeclampsia due to the improper exposure of the woman to the paternal sperm cells. Besides, one would also expect that the male factor group could cause implantation failure and early pregnancy losses more frequently due to the lack of the sperm cells leading defective inflammatory reaction in the endometrium. 

## METHODS

In this study, the medical records of 508 infertile patients were evaluated retrospectively who were admitted to our IVF center between the years January 2001 and March 2008 and had positive pregnancy test results after the ICSI procedure. Primary infertile cases without any medical disease were included in the study, whereas the patients older than 40 years of age, the patients with secondary infertility or with concomitant chronic hypertension, renal disease, type 1 diabetes were excluded. All pregnancies had ICSI procedure. The medical records of the patients regarding the infertility etiology, pregnancy detection rates, abortus rate, maternal age were collected from the database of the IVF center. Antenatal and maternal complications such as preeclampsia, placenta previa, placental abruption, premature rupture of membranes, premature labor, oligohydramnios, gestational diabetes, postmaturity, postpartum complications were evaluated via the file records and phone interviewing with the patients. Patients with preeclampsia were examined about the existence of the symptoms leading severe preeclampsia like headache, visual disturbance, upper abdominal pain, convulsion. The patients included in the study were divided into two main groups regarding the etiology of infertility: group one consisted of 301 infertile couples with male factor and group two patients were 207 infertile cases because of female factor and also unexplained infertility. Group one patients were divided further into two subgroups: group 1a included 56 cases in which TESE (testicular sperm extraction) was used to obtain the sperm cells as the male factor was severe. Group I b consists of 245 cases with obtaining sperm cells via conventional methods. procedures.

Statistical analyses were obtained through the NCSS 2007 package program. Chi-square test, t-test and odds ratio are used to compare the groups.

## RESULTS

The patients included in the study were divided into two main groups regarding the etiology of infertility: group one consists of 301infertile couples with male factor and group two patients were 207 infertile cases because of female factor (uterine factor, ovulatory factor, tubal factor) and also unexplained infertility. Group-1 patients were further divided into two subgroups: group 1a includes 56 cases in which TESE is used to obtain the sperm cells as the male factor was severe. Group-1b consisted of 245 cases with obtaining sperm cells via conventional procedures.

In Group-1, only 172 cases of 301 pregnancies passed over 24 weeks of gestational age, whereas in Group-2, 101 cases of 207 patients passed over 24 gestational weeks. There was no significant difference between two groups regarding chemical pregnancies and early pregnancy losses (p=0.314) (Table-I).

The mean age of women was 30.22±5.06 years in Group-1 and 31.58±4.36 years in group 2 respectively. The maternal age in Group-1 was significantly younger than that in Group-2 (p=0.001).

Regarding the Group-1 pregnancies passed over 24 weeks (n=170), 70.6% (120) of them were singletons, 24.1% (41) were twins and 5.3% (9) were triplets. Regarding the Group-2 pregnancies passed over 24 weeks (n=100), 73% (73) of them were singletons, 21.0% (21) were twins and 6% (6) were triplets. There was no difference between 2 groups concerning multiple pregnancies (p=0.829). 

Regarding the incidence of preeclampsia, there was no statistical important difference between Group-1 and Group-2 (p=0.328). However, there was 1.5 (0.67-3.22) times more risk for preeclampsia predisposition in Group-1 (Table-II). However, there was no significant difference between group 1a and group 1b regarding preeclampsia (p=0.825) (Table-II). When compared with Group-2, there was 1.3 fold risk for having preeclampsia in Group-1a (Table-II). Similarly, when compared with Group-2, there was 1.5 fold risk for having preeclampsia in Group-1b (Table-II).

There was no significant difference between group 1a, 1b and two regarding placenta previa, gestational diabetes, oligohydramnios, IUGR. About 12.6% of all pregnancies above 24 weeks gestational age of both groups were complicated with preeclampsia. The mean maternal age of preeclampsia was 32.38±4.2, and the mean maternal age of cases uncomplicated with preeclampsia was 29.84±4.28 (p=0.001). Regarding infertility etiology, there was not any significant difference between incidence of preeclampsia.

## DISCUSSION

Hypertension during pregnancy includes a wide variety of clinical spectrum. According to the classification of National High Blood Pressure Education on Working Group (2000), there are 5 subgroups of hypertensive disorders during pregnancy: preeclampsia, pregnancy induced hypertension, eclampsia, chronic hypertension, preeclampsia superimposed chronic hypertension.^11^ Regarding the pathophysiology of preclampsia, there are many hypothesis. Previously, preeclampsia was considered as simply a maternal disease with variable degrees of fetal involvement. Recent studies have showed some data about the unique immunogenetic maternal-paternal relationship. From that perspective, pre-eclampsia can be seen as a unique disease of eachdiscrete couple.^1^^,^^12^

**Table-I:** The main features of the study groups

	*Group I*	*Group II*	*P*
Mean maternal age (mean±SD)	30.22±5.06	31.58±4.36	0.001
Number of viable pregnancies >24 weeks	170	100	
Singletons	120 (70.6%)	73 (73.0%)	0.829
Twins	41 (24.1%)	21 (21.0%)	
triplets	9 (5.3%)	6 (6.0%)	

**Table-II:** Preeclampsia incidence regarding the study groups

	*ICSI with ejaculated sperm* *(group 1a)*		*ICSI with surgically obtained sperm* *(group 1b)*		*ICSI with female factor* *(group 2)*		*P♦*
	*N*	*%*	*n*	*%*	*n*	*%*	
Preeclampsia	3	12.5	21	9.9	10	9.9	0.825/ 0.713

*♦* *p in groups 1a vs. 1b and 1a vs. 2 respectively.*

Kyrou et al showed that there was no association between the etiology of infertility and preeclampsia.^13^With agreement of this study, we noticed that there was no statistical important difference between Group-1 and Group-2‚ in our study (p=0.328). However, there was 1.5 (0.67-3.22) times more risk for preeclampsia predisposition in Group-1.

It is still controversial how maternal immunity meets with paternal HLA antigens. An intermittent but long-lasting antigenic stimulus supported by appropriate cytokines like TGFβ1 is required to produce partner-specific mucosal immune tolerance.^14^ Antigenic factor lies on the spermatozoa cell, but not on sperm cells in seminal fluid prepared for insemination.^15^ Wang et al^16^ evaluated ICSI pregnancies in order to manifest the protective effect of sperm exposure and furthermore in order to investigate how this protective effect works; i.e. with sperm cell itself or with seminal fluid.

As there are no sperm cells in seminal fluid of patients with azospermia and as sperm cells are obtained surgically with testicular extraction, they are naturally an ideal model to test the protective partner-specific immune tolerance. In a study with 1621 patients, regarding the ICSI pregnancies with male factor, preeclampsia was 3-fold more common in pregnancies with surgically obtained sperm cells compared with that of conventionally obtained sperm cells.^16^ However, in our study, there was no statistically important difference between these 2 groups (group 1a and 1b) (p=0.825). Nevertheless, preeclampsia was 1.5-fold (0.6-3.3) more common with male factor compared with female factor and unexplained infertility. 

One of the largest studies comparing donor and partner inseminations includes 713 pregnancies, 438 of them with donor insemination and 275 of them with partner insemination. Singleton and multiple pregnancies complicated with preeclampsia were similar in both groups. However, preeclampsia was more common in donor insemination group (OR 3.7; CI 95%; 0.8-7.8).^13^From that perspective, repetitiveICSI cycles seem to be protective against preeclampsia (p=0.03).^13^

Our study had some limitations. The main limitation was the retrospective design of the study. The second unfavorable part of our study was that the patients with preeclampsia regarding all subgroups were older than the patients without preeclampsia. It may have an adverse effect to evaluate the sperm alloimmunisation situation.

Furthermore, regarding the singleton pregnancies, it is worth mentioning that the incidence of preeclampsia was 13.33% in pregnancies with male factor, which is much more higher than in general population with incidence of 2% to 7%.^2^In many studies so far, it is shown that preeclampsia was more often in IVF/ICSI pregnancies independent of the etiology of infertility.^17^Tabsh et al evaluated 116 IVF singletons and 27551 spontaneous singletons in a semi-prospective study for 5 years. Incidence of preeclampsia was 2.59% in IVF pregnancies, whereas 0.45% in spontaneous pregnancies (p<0.01).^17^In an another study of Shevell et al, 34.286 spontaneous singletons, 1222 singletons with ovulation induction and 554 IVF singletons are included. Preeclampsia incidence was 2.7 times more often in IVF pregnancies compared with that of spontaneous ones (CI:95%,1.7-4.4).^18^ The cause of higher incidence of preeclampsia in IVF pregnancies is still controversial: is it due to immunologic or medical co-morbidities unique to infertile couple or IVF procedure itself?.^19^ Genbacev et al proposed that the etiopathogenesis of preeclampsia and idiopathic infertility were very similar. IVF procedure itself and laboratory methods may be a predisposing factor for preeclampsia because the critical point for developing preeclampsia is the unsufficient development of early trophoblasts.^19^ Being primiparous most of the cases must also be kept in mind as a predisposing factor.^17^

## CONCLUSION

Our preliminary results show that the pregnancies with azoospermic partners had an increased risk for developing preeclampsia. Further studies are needed to confirm the paternal contribution in preeclampsia.

## Authors Contribution:

Burcu Artunc Ulkumen: Designed the research protocol and literature search.

Dilek B. Silfeler and Kenan Sofuoglu: Conducted the study and data analysis.

Ibrahim Silfeler and Vedat Dayicioglu: Prepared the final manuscript for publication.

## Notes

***Conflict interest: ***No conflict interest.
